# Issues in the Differential Diagnosis of Uterine Low-grade Endometrioid Carcinoma, Including Mixed Endometrial Carcinomas: Recommendations from the International Society of Gynecological Pathologists

**DOI:** 10.1097/PGP.0000000000000512

**Published:** 2018-12-14

**Authors:** Joseph T. Rabban, C. Blake Gilks, Anais Malpica, Xavier Matias-Guiu, Khush Mittal, George L. Mutter, Esther Oliva, Vinita Parkash, Brigitte M. Ronnett, Paul Staats, Colin J.R. Stewart, W. Glenn McCluggage

**Affiliations:** Pathology Department, University of California San Francisco, San Francisco, California (J.T.R.); Department of Pathology and Laboratory Medicine, Vancouver General Hospital and University of British Columbia, Vancouver, British Columbia, Canada (C.B.G.); Department of Pathology, The University of Texas MD Anderson Cancer Center, Houston, Texas (A.M.); Department of Pathology, University Hospital Arnau de Vilanova and Department of Pathology, University Hospital de Bellvitge, University of Lleida, IRBLLEIDA, IDIBELL, CIBERONC, Spain (X.M.G.); Department of Pathology, New York University Langone Medical Center, New York, New York (K.M.); Department of Pathology, Brigham and Women’s Hospital (G.L.M.); Pathology Department, Massachusetts General Hospital (E.O.), Boston, Massachusetts; Pathology Department, Yale New Haven Hospital, New Haven, Connecticut (V.P.); Department of Pathology, Johns Hopkins Medical Institutions (B.M.R.); Department of Pathology, University of Maryland School of Medicine (P.S.), Baltimore, Maryland; Department of Histopathology, King Edward Memorial Hospital and School for Women’s and Infants’ Health, University of Western Australia, Perth, Australia (C.J.R.S.); Department of Pathology, Royal Group of Hospitals Trust, Belfast, Northern Ireland, UK (W.G.M.)

**Keywords:** Endometrial cancer, Atypical hyperplasia, Serous carcinoma, Mixed carcinoma

## Abstract

This article provides practical recommendations developed from the International Society of Gynecological Pathologists Endometrial Carcinoma Project to address 4 issues that may arise in the diagnosis of uterine corpus low-grade endometrioid carcinoma: (1) The distinction between atypical hyperplasia and low-grade endometrioid carcinoma. (2) The distinction between low-grade endometrioid carcinoma and serous carcinoma. (3) The distinction between corded and hyalinized or spindle cell variants of low-grade endometrioid carcinoma and carcinosarcoma. (4) The diagnostic criteria for mixed endometrial carcinomas, a rare entity that should be diagnosed only after exclusion of a spectrum of tumors including morphologic variants of endometrioid carcinoma, dedifferentiated endometrial carcinoma, carcinosarcoma, and endometrial carcinomas with ambiguous morphology.

Approximately 80% of endometrial carcinomas are of endometrioid type and most of these are morphologically low grade (International Federation of Gynecology and Obstetrics [FIGO] grade 1 or 2). A variety of problems may occur in the diagnosis of low-grade endometrioid carcinomas; some of these are discussed herein and practical recommendations to aid in diagnosis are provided. These recommendations were developed by the authors as part of the International Society of Gynecological Pathologists Endometrial Carcinoma Project.

## DISTINCTION BETWEEN LOW-GRADE ENDOMETRIOID CARCINOMA AND ATYPICAL HYPERPLASIA

### Recommendations

1. The presence of glandular crowding with endometrial stromal exclusion and significant cribriform, confluent glandular, labyrinthine, papillary/villoglandular, or nonsquamous solid architecture distinguishes low-grade endometrioid carcinoma from atypical hyperplasia. These features may be present alone or in combination.
2. The definition of the minimum amount (size of span, surface area, or number of fragments) of these patterns needed to diagnose low-grade endometrioid carcinoma is not resolved and remains a subjective interpretation by the individual pathologist in an individual case.
3. If the morphologic features are suspicious but do not fully meet the criteria for endometrioid carcinoma, this concern should be communicated descriptively in the pathology report rather than being classified as atypical hyperplasia without further comment.
4. There are no diagnostically useful biomarkers to distinguish between atypical hyperplasia and low-grade endometrioid carcinoma.

### Discussion

**FIG. 1:**
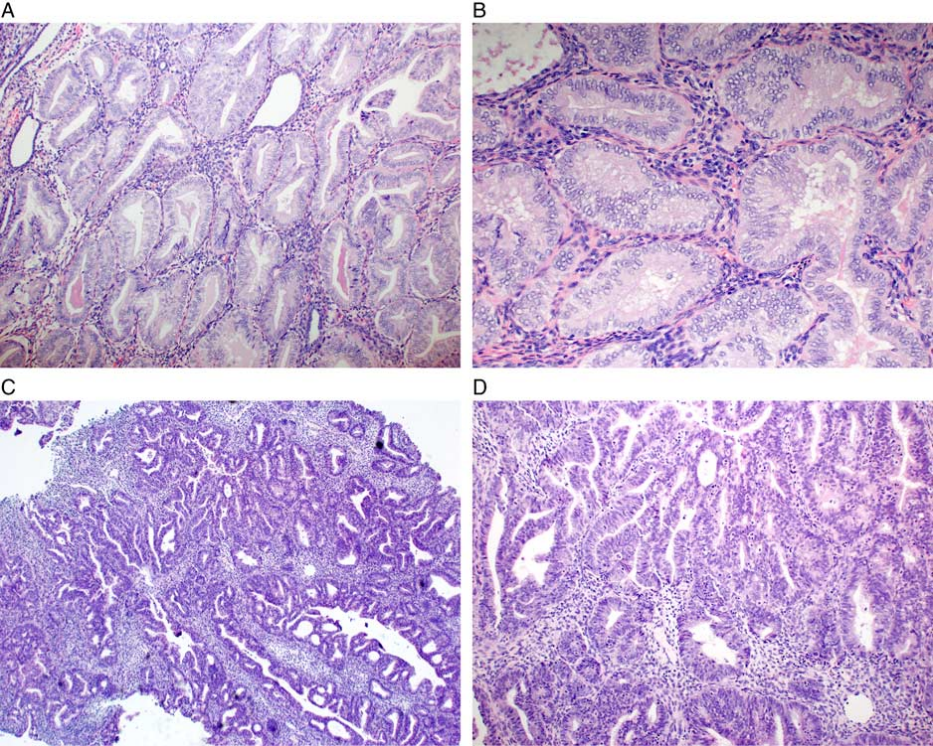
In endometrial atypical hyperplasia, the glands are crowded but not confluent (A) and endometrial stroma is preserved around the glands (B). On occasion, the presence of small foci suggestive of confluent architecture (C, D) within atypical hyperplasia may raise suspicion for small foci of grade 1 endometrioid carcinoma but may not be interpreted to meet the criteria for a definite diagnosis of malignancy. The interpretation of such cases can be problematic and subject to interobserver variation, especially since there are no evidence-based guidelines for the minimum size of confluent growth that predicts myoinvasive endometrioid carcinoma. If the findings are not deemed to meet criteria for cancer, it is recommended to report the diagnosis as atypical hyperplasia with features suspicious for grade 1 endometrioid carcinoma (or using equivalent wording).

**FIG. 2:**
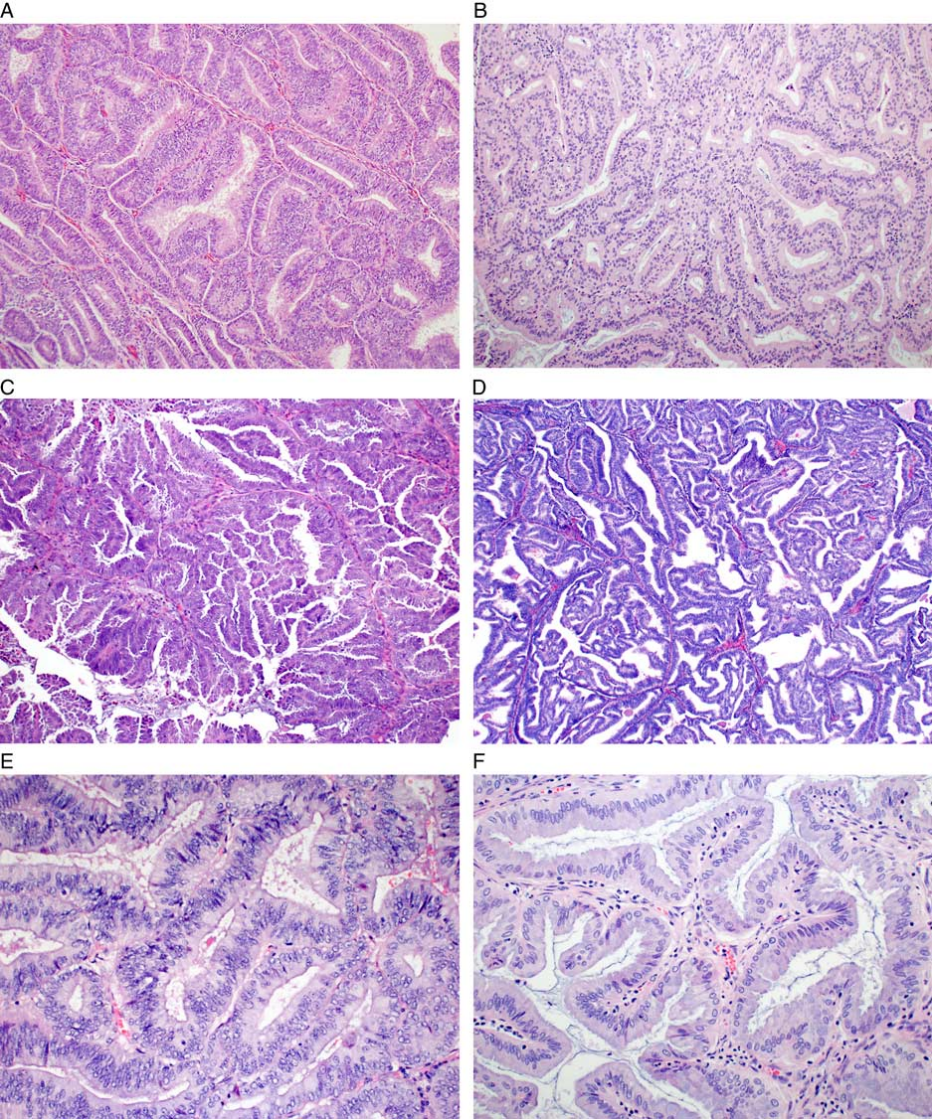
Architectural patterns that meet qualitative criteria for classifying an endometrial proliferation as grade 1 endometrioid carcinoma: confluent glandular growth (A); cribriform growth (B); confluent papillary growth (C); complex labyrinthine growth (D). There is no evidence-based consensus on the minimum size of these patterns needed to classify as endometrioid carcinoma. Absence of endometrial stroma between the glands is required for classification as endometrioid carcinoma (E); however there may be other cell types in between the glands such as lymphocytes, neutrophils, plasma cells, histiocytes, and/or endothelium of blood vessels (F).

The standard management of atypical hyperplasia (synonymous terminology is endometrioid intraepithelial neoplasia) (Fig. 1) is total hysterectomy whereas additional surgical staging may be considered for low-grade endometrioid carcinoma (Fig. 2) 1,2. Thus, setting aside nonsurgical options for women wishing to preserve fertility, there is clinical merit in attempting to distinguish atypical hyperplasia from low-grade endometrioid carcinoma in preoperative endometrial samples.

Proposed criteria for defining low-grade endometrioid carcinoma are largely drawn from studies designed to identify morphologic features in an endometrial biopsy or curettage that predict myoinvasive endometrioid carcinoma in a subsequent hysterectomy 3–5. Key predictors include glandular crowding with endometrial stromal exclusion and significant cribriform, confluent glandular, labyrinthine, papillary/villoglandular, or nonsquamous solid architecture (Fig. 2). The minimal amount of such findings that qualify for a diagnosis of carcinoma have been studied only to a limited degree and the conclusions depend on the desired combination of sensitivity and specificity for predicting myoinvasive cancer. Proposed minimum thresholds to define cancer include a size of 2.1 mm of any single fragment, an aggregate size of at least 3 mm, whether a single fragment or multiple smaller fragments 5, or 30% volume of the sample involved by these features 4. Notably, at least 1 study reported that the presence of any amount of cribriform growth regardless of size was predictive of myoinvasive cancer 5. Presently, there is not enough evidence to define a specific minimum amount of any of these growth patterns and so the interpretation is left to the individual pathologist in each individual case. On occasion, the features in a biopsy may be at the cusp between atypical hyperplasia and grade 1 endometrioid carcinoma without fully meeting criteria to classify outright as cancer (Figs. 1C, D). At least 1 study demonstrated that these patients have a higher incidence of cancer in the subsequent hysterectomy compared with patients whose biopsy exhibits atypical hyperplasia without any concerning features for cancer 6. This suggests that such cases should not be reported merely as a conventional atypical hyperplasia but that the strong suspicion for cancer should be discussed in the pathology report.

The proposed architectural criteria for cancer are most predictive in samples that contain adequate, intact tissue and are challenging to apply in samples where the amount of tissue is limited in size or the tissue is highly fragmented or disrupted. Diagnostic subjectivity in assessing these qualitative criteria may also contribute to discordance between biopsy and hysterectomy findings and discordance between observers in classifying biopsy and/or hysterectomy features 7,8.

There are no immunohistochemical markers that facilitate the distinction between atypical hyperplasia and low-grade endometrioid carcinoma.

## DISTINCTION BETWEEN LOW-GRADE ENDOMETRIOID CARCINOMA AND SEROUS CARCINOMA

### Recommendations

1. The morphologic concordance/discordance between glandular/papillary architecture and nuclear atypia distinguishes most low-grade endometrioid carcinomas from glandular and papillary patterns of serous carcinoma. The former typically exhibits concordant low-grade architecture and low-grade nuclear atypia. The latter exhibits discordance of these features, with low-grade architecture but high-grade nuclear atypia. Because of the differences in surgical management of these tumor types, attention to the nuclear features is of particular importance in biopsy and frozen section specimens containing a tumor exhibiting low-grade glandular architecture.
2. Secondary features that favor endometrioid carcinoma are coexistent endometrial hyperplasia, squamous, mucinous, and/or other forms of differentiation, polarity of nuclei with a well-defined luminal border, and microcystic elongated and fragmented pattern of myoinvasion. Secondary features that favor serous carcinoma are so-called serous endometrial intraepithelial carcinoma, loss of nuclear polarity with detached and budding tumor cells, serrated or slit-like intraluminal gland contours, and so-called “gaping gland” pattern of myoinvasion. Serous carcinoma should be considered if the tumor arises in atrophic endometrium or in an endometrial polyp, although these features are not specific.
3. Careful evaluation of tumor morphology reliably distinguishes most cases of low-grade endometrioid carcinoma from serous carcinoma. In those cases where there is morphologic doubt and where further confirmation is required, the best marker is p53; aberrant (mutation-type) staining (either the overexpression pattern or null pattern) favors serous carcinoma, whereas wild-type staining favors endometrioid carcinoma. p16 is also useful, with diffuse block-type immunoreactivity favoring serous carcinoma. The results of these markers should always be interpreted in conjunction with evaluation of the tumor morphology, keeping in mind that rare exceptions exist and that even non-neoplastic lesions, such as papillary syncytial metaplasia, can exhibit diffuse p16 staining.

### Discussion

Some serous carcinomas may mimic low-grade endometrioid carcinoma, especially on low-power magnification 9–12. The distinction between these 2 neoplasms is essential as the surgical and adjuvant management are vastly different 2.

**FIG. 3:**
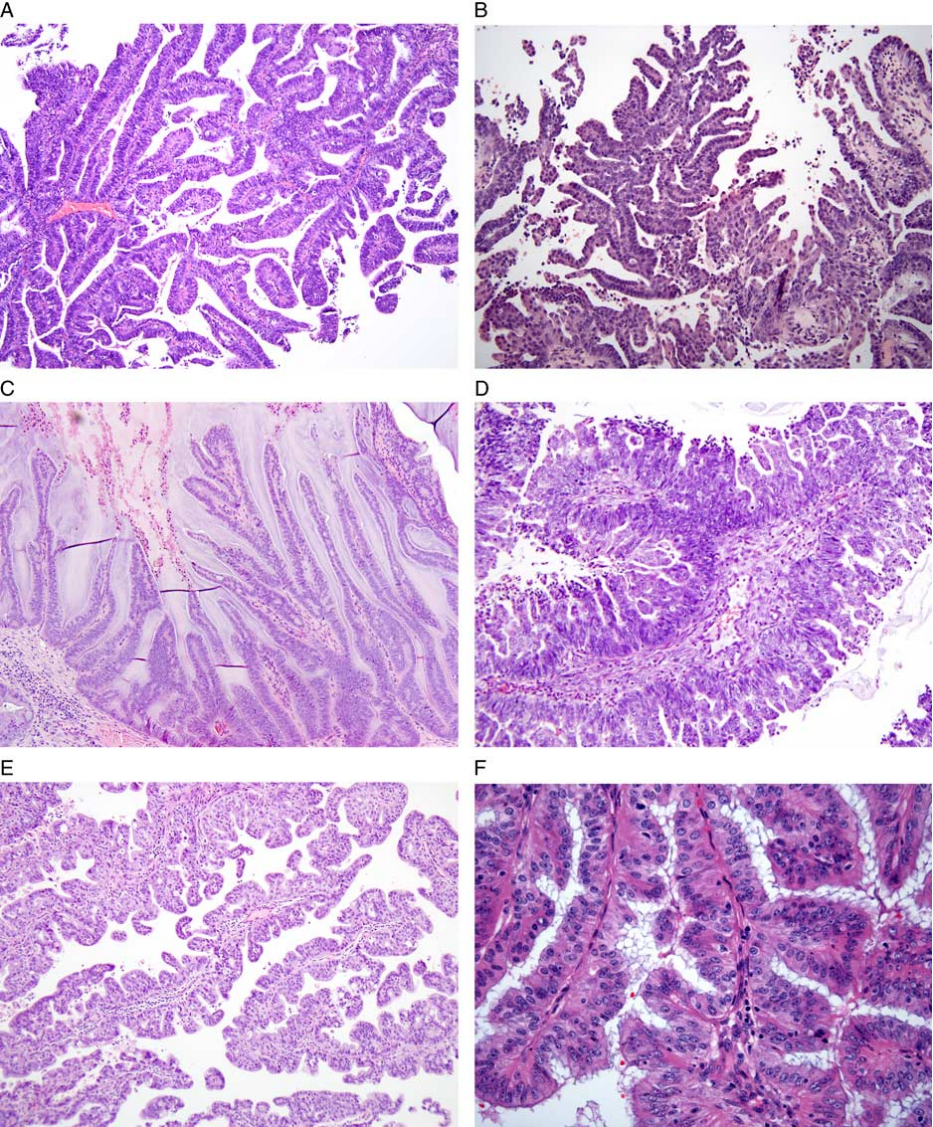
Low magnification patterns of low-grade endometrioid carcinoma that may be misinterpreted as serous carcinoma include papillary (A), micropapillary (B), villoglandular (C), and with small non-villous papillae (D, E). Unlike serous carcinoma, the nuclei exhibit minimal atypia and low mitotic activity (F).

Two variations of low-grade endometrioid carcinoma, 1 of which is architectural and 1 of which is cytologic, may resemble serous carcinoma. First, papillary, villoglandular, and small nonvillous papillary architecture may be present, raising concern for the prototypical papillary architecture of serous carcinoma (Fig. 3) 9,13. However, cytologic features of serous carcinoma are not present in these architectural variants; there is generally no or minimal nuclear hyperchromasia and an absence of eosinophilic macronucleoli, significant nuclear atypia, and atypical mitoses. The presence of villoglandular and/or papillary patterns in what is otherwise a typical low-grade endometrioid carcinoma does not affect the tumor behavior 13. These patterns, in the absence of severe nuclear atypia, should be considered as analogs to the glandular pattern of endometrioid carcinoma when determining FIGO grade. Thus, it is the concordance between low-grade architecture and low-grade cytology that permits distinction of the villoglandular and papillary variants of low-grade endometrioid carcinoma from serous carcinoma 11. Additional features that support classification as endometrioid carcinoma are squamous, mucinous, and/or other forms of differentiation (eg, eosinophilic metaplastic-type differentiation), endometrial hyperplasia and, in a hysterectomy specimen, the microcystic elongated and fragmented pattern of myometrial invasion.

A second setting in which low-grade endometrioid carcinoma may raise a differential diagnosis with serous carcinoma is when there is focal or diffuse moderate nuclear atypia and/or increased mitotic activity at the higher end of the spectrum of what is generally observed in endometrioid carcinoma exhibiting low-grade architecture. The generally focal nature of any moderate nuclear atypia and the lack of diffuse severe atypia favor classification as endometrioid carcinoma, as does the presence of squamous/mucinous differentiation and/or endometrial hyperplasia. Although mitotic activity is generally much lower in low-grade endometrioid carcinoma than in serous carcinoma, there are no studies that identify a specific mitotic index that reliably distinguishes the two tumor types from each other 14.

**FIG. 4:**
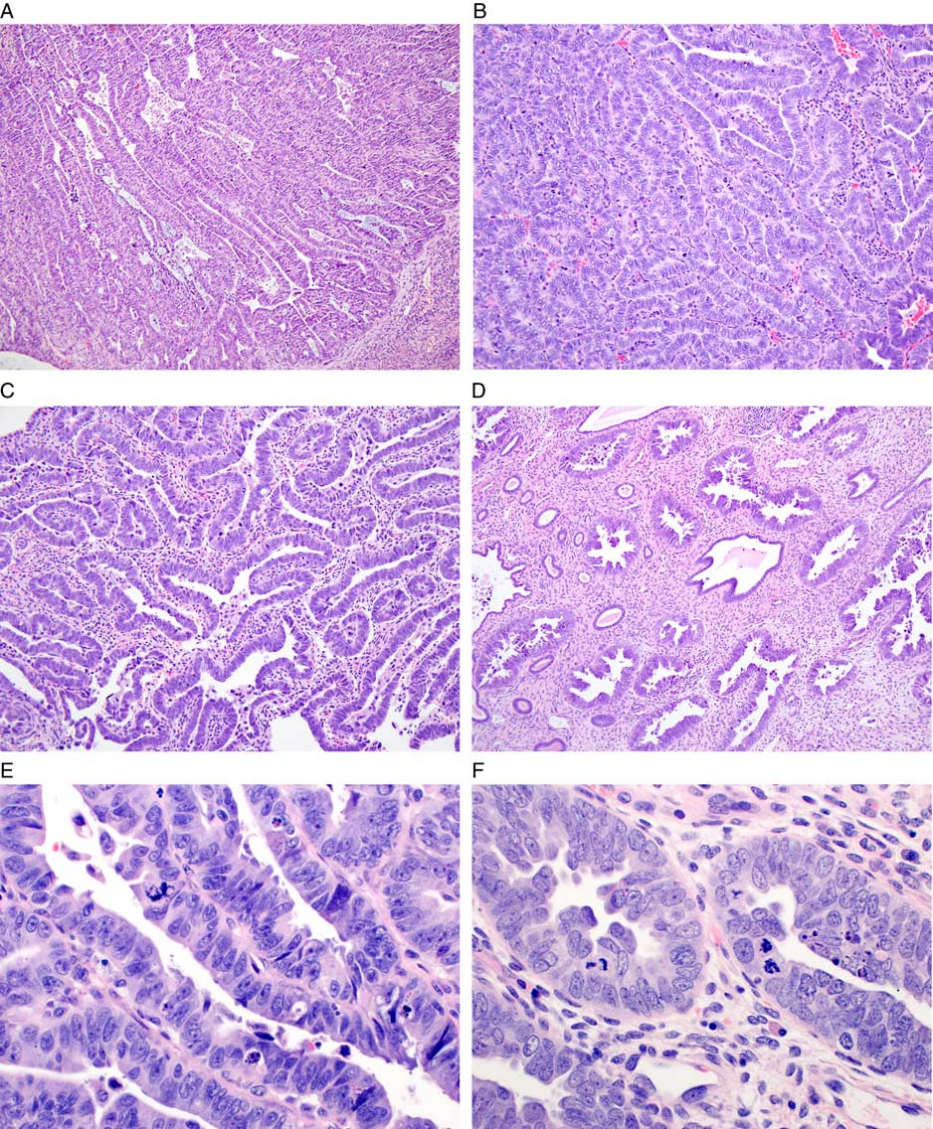
The glandular pattern of endometrial serous carcinoma may mimic the architecture of low-grade endometrioid carcinoma at low (A) and medium (B) magnification. Serous carcinoma may also mimic atypical hyperplasia (C), particularly within an endometrial polyp (D). However, at high magnification (E, F), serous carcinoma exhibits nuclear pleomorphism, high nuclear to cytoplasmic ratio, macronucleoli, high mitotic activity, and atypical mitotic figures. None of these features are expected in a low-grade endometrioid carcinoma (Fig. 2E and F).

Conversely, a glandular pattern of serous carcinoma may simulate low-grade endometrioid carcinoma or even atypical endometrial hyperplasia; the latter may be particularly problematic in endometrial polyps (Fig. 4) 10,12. At low magnification, the entire tumor may exhibit simple tubulo-glandular architecture, without any prototypical architecture of serous carcinoma such as papillary or solid growth. However, at high magnification, the tumor cells show classical cytologic features of severe nuclear atypia, hyperchromasia, eosinophilic macronucleoli, high mitotic index, and atypical mitotic figures. Thus, the discordance between the low-grade architecture and high-grade cytology usually permits distinction of this variant of serous carcinoma from low-grade endometrioid carcinoma 11,12. Secondary features of serous carcinoma include budding and detachment of the tumor cells and so-called “gaping gland” pattern of myoinvasion in hysterectomy specimens. Serous carcinoma often arises in a background of atrophic endometrium or in an endometrial polyp; these features alone are not specific to serous carcinoma but should raise consideration of this tumor type. So-called serous endometrial intraepithelial carcinoma, defined as replacement of the existing endometrial glands by malignant cells exhibiting serous morphology and immunophenotype, is sometimes present adjacent to prototypical serous carcinoma but is not present in a low-grade endometrioid carcinoma.

**FIG. 5:**
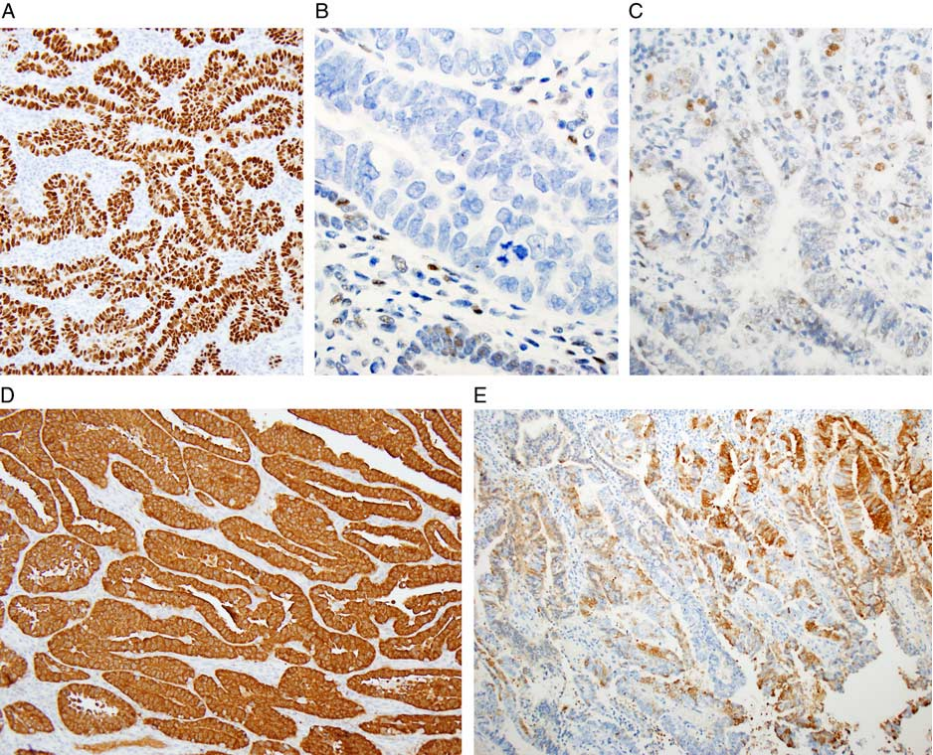
Mutation-type p53 immunoexpression in endometrial serous carcinoma is defined as either diffuse strong nuclear staining in >80% of tumor cells (A) or complete absence of any staining in tumor cells (B). In the latter setting, weak patchy p53 staining of lymphocytes, stroma and normal endometrial glands serves as a positive internal control. p53 staining in grade 1 endometrioid carcinoma is weak and patchy (wild-type immunoreactivity) (C). p16 staining in serous carcinoma is typically diffuse and strong (D) and is typically patchy in grade 1 endometrioid carcinoma (E).

Careful evaluation of tumor morphology is sufficient to distinguish most cases of low-grade endometrioid carcinoma from serous carcinoma. In the occasional setting in which immunohistochemistry is needed to confirm the morphologic interpretation, p53 is the best marker (Fig. 5) 12,15–20. Aberrant (mutation-type) staining is present in virtually all serous carcinomas, whereas wild-type staining is present in almost all low-grade endometrioid carcinomas, although a small proportion of the latter exhibit mutation-type immunoreactivity. In the setting of equivocal p53 results, p16 is also useful as it is diffusely positive in a very high proportion of serous carcinomas, whereas low-grade endometrioid carcinomas usually exhibit patchy so-called “mosaic-type” staining (Fig. 5) 21,22. Even when most of an endometrioid carcinoma is positive, there are typically alternating positive and negative foci without diffuse block-type immunoreactivity. Caution is advised against interpreting the results of markers in isolation of the morphologic findings since there are always occasional exceptions to the typical staining patterns and even non-neoplastic lesions, such as papillary syncytial metaplasia of the endometrium, can be diffusely positive for p16 23. While other markers may be of value, there is significant overlap. In particular, many serous carcinomas are focally or diffusely positive with estrogen receptor and this marker is of limited value in distinction from a low-grade endometrioid carcinoma. Other markers, such as PTEN, which shows complete loss in a subset of endometrioid carcinomas but not in serous carcinoma, may be of some value; however, this marker is not widely available, can be technically difficult to perform and there can be problems in interpretation 12,24.

## DISTINCTION BETWEEN CARCINOSARCOMA AND ENDOMETRIOID CARCINOMA WITH CORDED AND HYALINIZED OR SPINDLE CELL PATTERNS.

### Recommendations

1. The degree of nuclear atypia generally distinguishes endometrioid carcinoma with corded and hyalinized pattern or spindle cell features from carcinosarcoma. The epithelial and mesenchymal components of carcinosarcoma typically exhibit severe nuclear atypia, mitotic activity and, often, atypical mitotic figures. In contrast, these variants of endometrioid carcinoma typically exhibit low-grade atypia. While heterologous elements are often present in carcinosarcoma, bland-appearing osteoid and chondroid formation may rarely occur in these low-grade variants of endometrioid carcinoma.
2. In carcinosarcoma, the epithelial and mesenchymal components are usually clearly spatially distinct from each other without directly blending together. In contrast, the spindle cell and corded and hyalinized components typically seamlessly merge with the conventional glandular component of endometrioid carcinoma. However, there are exceptions in that occasionally there is a sharp demarcation between the spindle cell and corded and hyalinized components and the conventional glandular component.
3. p53 immunohistochemistry may be of value to help confirm a diagnosis of carcinosarcoma as both the epithelial and mesenchymal components often exhibit mutation-type immunoreactivity, in contrast to wild-type staining in most cases of low-grade endometrioid carcinoma. Epithelial markers, such as epithelial membrane antigen (EMA) and broad spectrum cytokeratins, are often positive at least focally in the spindle cell and corded components of endometrioid carcinoma but are generally not expressed (or are expressed to a limited extent) in the mesenchymal component of carcinosarcoma.
4. Grading of corded/hyalinized and spindle cell variants of endometrioid carcinoma should be based only on the conventional glandular component of endometrioid carcinoma, although it is acknowledged that evidence-based criteria for grading in this setting do not exist. Carcinosarcoma should be considered a high-grade tumor.

### Discussion

**FIG. 6:**
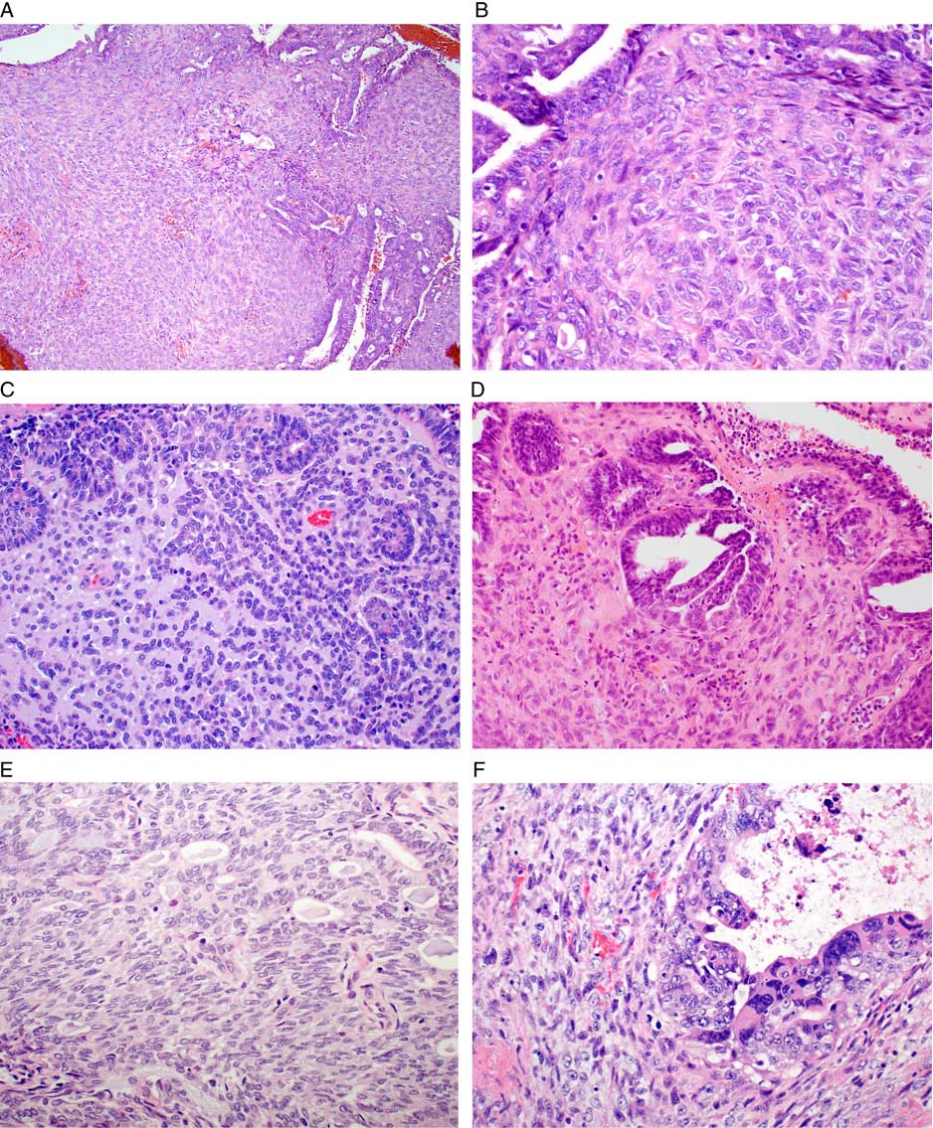
Corded and hyalinized endometrioid carcinoma (A–D). Spindled and oval tumor cells grow in a streaming (A), corded (B, C) or sex-cord like pattern within myxoid (C) and/or hyaline (B, D) stroma. Solid growth of spindled cells merge with glandular areas of endometrioid carcinoma (E). These variants of low-grade endometrioid carcinoma exhibit low-grade nuclear atypia in contrast to carcinosarcoma (F), in which both the glandular and mesenchymal components exhibit severe nuclear atypia.

Two variants of low-grade endometrioid carcinoma may morphologically mimic carcinosarcoma. These are endometrioid carcinoma with spindle cell formation and corded and hyalinized endometrioid carcinoma; these are likely related neoplasms with some cases exhibiting overlapping morphologic features (Fig. 6) 25–27. Outcome data are limited for these uncommon variants of endometrioid carcinoma but it appears that the presence of these variant growth patterns does not alter the behavior that would be predicted by the grade of the conventional component of endometrioid carcinoma and the stage; these are usually, but not always, low-stage neoplasms 25,26. Although there are no evidence-based studies regarding grading these variants of endometrioid carcinoma, the consensus approach is to base the grade only on evaluation of the conventional component of endometrioid carcinoma, which is FIGO grade 1 or 2 in most cases. For these reasons, these variants should not be interpreted as carcinosarcoma, which they may morphologically resemble given their biphasic appearance. In contrast to these variants, carcinosarcoma is considered to be a high-grade endometrial cancer 2,28.

Spindle cell features may be present in endometrioid carcinoma of the endometrium or, more commonly, the ovary 25,26. The tumor cells grow in vague fascicles, bundles, or nests. They exhibit spindle cell shape with indistinct cytoplasmic borders. The areas of spindle cells usually blend seamlessly with adjacent areas of more conventional endometrioid carcinoma which is usually low grade. The degree of nuclear atypia is usually mild to moderate in both the spindle cell and conventional glandular areas of tumor; mitotic activity is also usually relatively low in the spindled and glandular components. The spindle cell elements may resemble morules or solid squamous differentiation, and in some cases there may be areas of squamous differentiation, including keratinization. The spindle cells usually exhibit epithelial differentiation by immunohistochemistry with positive staining with EMA and broad spectrum cytokeratins, although the degree of immunoreactivity is generally less than in the glandular component; p53 exhibits wild-type immunoreactivity.

Spindle cell features may also be observed in corded and hyalinized endometrioid carcinoma, which is an endometrioid carcinoma containing cord-like growth of low-grade epithelioid or spindle cells within a hyaline or myxoid stroma 25. The cords may interanastomose or may form small clusters. Discrete foci of keratinizing squamous differentiation may occasionally be present within the hyaline or myxoid stroma. The corded areas are usually admixed with a conventional glandular component of endometrioid carcinoma and the 2 components usually seamlessly blend together, although occasionally there is a sharp demarcation. The corded tumor cells exhibit epithelial differentiation by immunohistochemistry, although the extent of expression of epithelial markers (EMA and broad spectrum cytokeratins) is usually less than in the admixed glandular tumor component. Heterologous osseous and chondroid differentiation may be present, although the nuclear features are bland.

Two morphologic features permit distinction of these variants of endometrioid carcinoma from carcinosarcoma 29. First, these variant patterns do not usually exhibit a sharp demarcation from the areas of conventional endometrioid carcinoma, as is usual for the mesenchymal and epithelial components of carcinosarcoma. Instead, the variant patterns and glandular patterns typically merge seamlessly together, although, as discussed, there are occasional exceptions. Second, the spindle cell and corded elements do not exhibit severe nuclear atypia, high mitotic index, or atypical mitoses, which would be expected in the mesenchymal component of carcinosarcoma. When heterologous osseous or chondroid elements are present in corded and hyalinized endometrioid carcinoma, the nuclear features are bland, unlike the usual malignant features of the heterologous elements in carcinosarcoma. Epithelial differentiation by immunohistochemistry and wild-type p53 immunoreactivity are present in spindle cell or corded and hyalinized variants of endometrioid carcinoma 25,26. Conversely, the mesenchymal component of carcinosarcoma exhibits limited or absent expression of epithelial markers and, along with the epithelial component, which often exhibits serous differentiation, commonly shows aberrant (mutation-type) p53 staining 30–33.

## CRITERIA FOR MIXED ENDOMETRIAL CARCINOMA

### Recommendations

1. Mixed endometrial carcinoma is composed of 2 or more spatially distinct tumor subtypes, at least 1 of which is serous carcinoma or clear cell carcinoma. The most common combination is an admixture of endometrioid carcinoma and serous carcinoma. This term does not apply to morphologic variations of endometrioid carcinoma, serous carcinoma, or clear cell carcinoma, nor does it apply to endometrioid carcinoma admixed with mucinous carcinoma, endometrial carcinoma with ambiguous morphology, dedifferentiated endometrial carcinoma or carcinosarcoma.
2. Any amount of serous carcinoma or clear cell carcinoma that can be confidently recognized on routine hematoxylin and eosin–stained sections in an otherwise endometrioid carcinoma qualifies for a mixed carcinoma. It is acknowledged that an evidence-based definition does not exist for the minimal amount of serous carcinoma or clear cell carcinoma admixed with endometrioid carcinoma that carries clinical significance.
3. Immunohistochemistry to confirm the diagnosis of each component of tumor is advised, using the immunostains typically positive (and negative) in each subtype, as discussed elsewhere in the ISGyP recommendations.
4. The pathology report should include a list of each tumor type (and grade) and their percent composition. However, the tumor should be graded overall as high grade (grade 3) regardless of the relative amount of serous or clear cell carcinoma present.

### Discussion

**FIG. 7:**
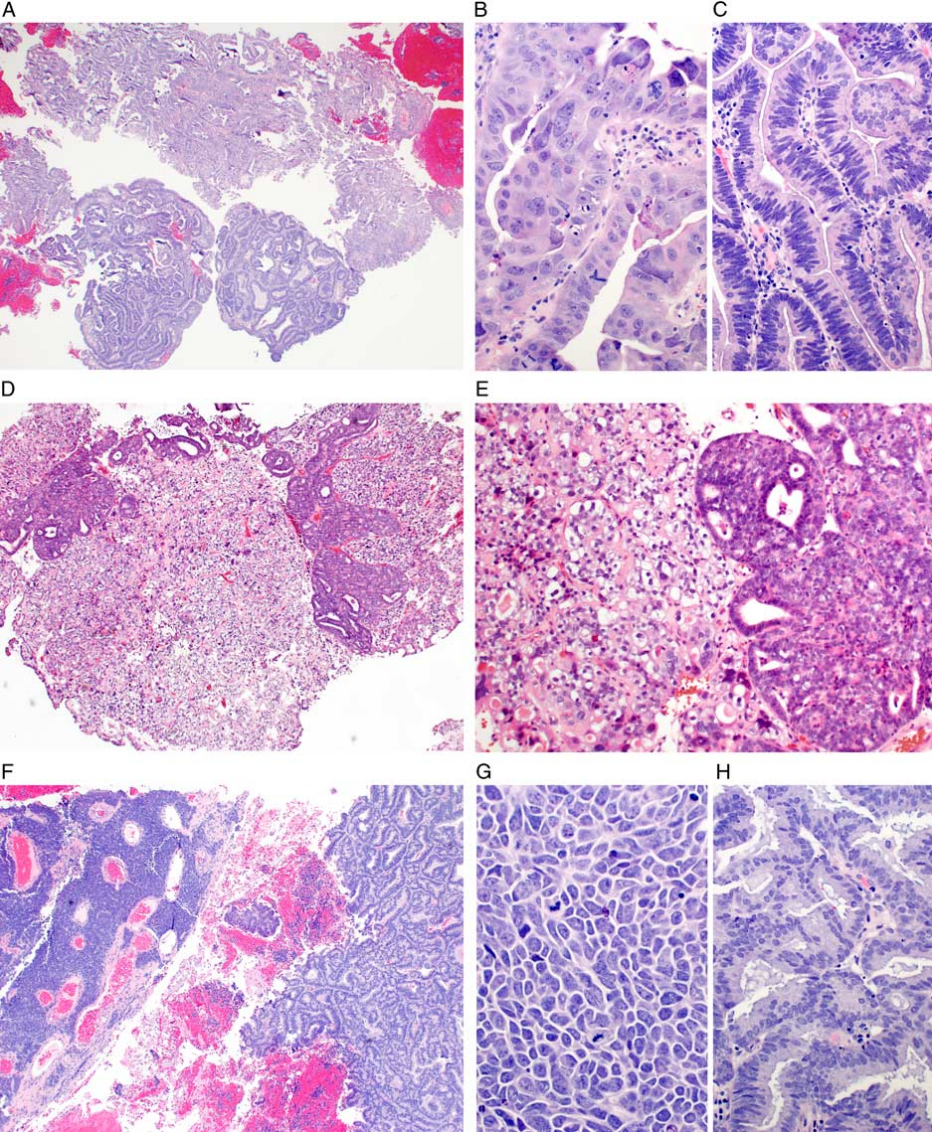
Biopsies of mixed endometrial carcinomas. Each contains a spatially distinct component of low-grade endometrioid carcinoma but the high grade component defines the behavior and management. Mixed serous carcinoma and endometrioid carcinoma (A–C). B is a high-magnification view of the serous component and C of the endometrioid component. Mixed clear cell carcinoma and endometrioid carcinoma (D, E). Mixed high grade neuroendocrine carcinoma and endometrioid carcinoma (F–H); G is a high-magnification view of the neuroendocrine component and H of the endometrioid component.

The definition of mixed endometrial carcinoma has evolved as the overall classification scheme for endometrial carcinomas has been refined. The 2014 World Health Organization (WHO) classification defines mixed endometrial carcinoma as a tumor composed of 2 or more spatially distinct tumor subtypes, at least one of which is serous carcinoma or clear cell carcinoma (Fig. 7) 28. The most common mixed endometrial carcinoma is a mixture of endometrioid carcinoma and serous carcinoma. Mixed carcinomas with a component of neuroendocrine carcinoma also occur but these are not discussed further. The rationale for recognizing mixed endometrial carcinoma is that even a minor component of serous or clear cell carcinoma within an otherwise typical endometrioid carcinoma may confer an adverse outcome similar to a pure serous or clear cell carcinoma. Importantly, this entity is a diagnosis of exclusion. First, the term should not be used for pathologically distinct tumor types, such as dedifferentiated endometrial carcinoma or carcinosarcoma 29,34,35. Second, the term should not be used for morphologic variants of endometrioid carcinoma that simulate serous carcinoma (eg, villoglandular or papillary variants of endometrioid carcinoma) or clear cell carcinoma (eg, clear cell change in endometrioid carcinoma) 9. Finally, the term should not be used for cases with ambiguous morphology that defy classification 36. Thus, as a diagnosis after exclusion of a number of other tumors, true cases of mixed endometrial carcinoma are exceedingly rare. Not surprisingly, mixed endometrial carcinoma remains a common source of interobserver disagreement in endometrial cancer subtyping 37–41.

Two modifications to the WHO criteria for mixed endometrial carcinoma are advised. First, immunohistochemistry is recommended to help confirm the subtype of each component, using the suggested markers (positive and negative) for each individual subtype. This strategy mitigates against misinterpreting morphologic variations of endometrioid carcinoma, such as those with villoglandular, papillary, or clear cell features, as a separate component of serous or clear cell carcinoma. Second, it is advised that any amount of serous carcinoma or clear cell carcinoma that can be confidently recognized to coexist with endometrioid carcinoma qualifies as a mixed epithelial carcinoma. This strategy differs slightly from the WHO criterion which recommends that the serous or clear cell carcinoma component must comprise at least 5% of the overall tumor. The WHO recommendation was based on studies that used a 5% threshold for the minor component; however, there are no outcome studies that have addressed tumors that fall under that threshold. Until such studies are available, it is recommended not to use a specific quantitative threshold to exclude cases that clearly exhibit prototypical features of serous carcinoma or clear cell carcinoma.

The pathogenesis of mixed endometrial carcinoma remains to be fully elucidated. Recent molecular genetic studies suggest that some may represent collision tumors composed of genetically independent tumors while others may represent progression or divergence from a common origin with the serous or clear cell component deriving from the endometrioid carcinoma 42,43.

Two other endometrial carcinomas that contain spatially distinct components of tumor types are dedifferentiated endometrial carcinoma and carcinosarcoma. However, these are both pathologically distinct tumors that should not be classified as mixed endometrial carcinoma but as their own subtypes. Dedifferentiated endometrial carcinoma consists of an undifferentiated component (i.e. undifferentiated endometrial carcinoma) and a spatially distinct low-grade endometrioid component 28,34,35. Dedifferentiated endometrial carcinoma exhibits aggressive behavior even when the amount of undifferentiated endometrial carcinoma is minor 34,35,44,45. Mismatch repair gene defects and mutations of the switch/sucrose non-fermentable chromatin remodeling complex are common 46–49. Undifferentiated endometrial carcinoma typically appears as sheets of noncohesive monomorphic tumor cells that are negative or only focally positive for epithelial markers (broad spectrum cytokeratins, EMA), Mullerian markers (PAX8, estrogen receptor), and cell adhesion (E-cadherin) markers. Some undifferentiated endometrial carcinomas may also exhibit rhabdoid features, cord-like growth, or a myxoid stroma. Switch/sucrose non-fermentable complex component (SMARCA2, SMARCA4, SMARCB1, ARID1A, and/or ARID1B) deficiency and loss of mismatch repair protein expression are common immunohistochemical features of undifferentiated endometrial carcinoma and the dedifferentiated component of dedifferentiated endometrial carcinoma 46–49.

Carcinosarcoma contains a malignant epithelial component that may reflect various histologic types (eg, serous, endometrioid, clear cell) and a spatially distinct malignant mesenchymal component that may include heterologous differentiation 29. Carcinosarcoma is a distinct tumor type and should not be classified as mixed endometrial carcinoma.
